# Natural Orifice Translumenal Endoscopic Surgery for Anterior Spinal Procedures

**DOI:** 10.1155/2012/365814

**Published:** 2012-05-24

**Authors:** Priscilla Magno, Mouen A. Khashab, Manuel Mas, Samuel A. Giday, Jonathan M. Buscaglia, Eun Ji Shin, Xavier Dray, Anthony N. Kalloo

**Affiliations:** ^1^Division of Gastroenterology and Hepatology, University of Puerto Rico School of Medicine, San Juan, PR 00936, USA; ^2^Division of Gastroenterology and Hepatology, Johns Hopkins Hospital, 600 N. Wolfe Street, Blalock 465, Baltimore, MD 21287, USA; ^3^Department of Surgery, University of Puerto Rico School of Medicine, San Juan, PR 00936, USA; ^4^Department of Digestive Diseases, Lariboisière Hospital, AP-HP, Paris, France

## Abstract

*Background*. NOTES techniques allow transesophageal access to the mediastinum. The aim of this study was to assess the feasibility of transesophageal biopsy of thoracic vertebrae. *Methods*. Nonsurvival experiments on four 50-kg porcine animals were performed. Transesophageal access to the mediastinum was attained using submucosal tunneling technique. *Results*. The posterior mediastinum was successfully accessed and navigated in all animals. Vertebral bodies and intervertebral spaces were easily approached while avoiding damage to adjacent vessels. Bone biopsy was successfully performed without complications, but the hardness of bone tissue resulted in small and fragmented samples. *Conclusions*. Peroral transesophageal access into the posterior mediastinum and thoracic vertebral bone biopsy was feasible and safe. The proximity of the esophagus to the vertebral column provides close and direct access to the thoracic spine and opens up new ground for the performance of multilevel anterior spine procedures using NOTES techniques.

## 1. Introduction

Natural orifice transluminal endoscopic surgery (NOTES) has gained a great deal of attention from gastroenterologists and surgeons all over the world since its introduction in 2000 [1]. Interest in NOTES procedures within the thoracic cavity is gaining momentum [2–7].

Transesophageal approach into the mediastinum has been successfully performed in animal and cadaveric models via endoscopic full thickness incision of the esophageal wall, submucosal endoscopy techniques or assisted by endoscopic ultrasound (EUS) [7, 8]. Transesophageal NOTES enables access into the posterior mediastinum with visualization of the descending thoracic aorta, esophagus, trachea, pleura, lung, vagus nerves, and hilar lymph nodes [9]. The excellent visualization of these structures has allowed for a variety of transesophageal mediastinal NOTES interventions including mediastinal lymph node resection, vagotomy, thoracic duct ligation, thymectomy, biopsy of the lung and pleura, epicardial coagulation, saline injection into the myocardium, and pericardial fenestration [4, 5, 7].

Transesophageal NOTES is still in its infancy. However, its potential clinical applications deserve commitment from NOTES researchers to further investigate potential novel applications for transesophageal NOTES. The proximity of the esophagus to the vertebral column provides a closer and direct access to the thoracic spine and opens a new ground for multilevel anterior spine procedures using NOTES techniques. Furthermore, a NOTES approach to the spine could potentially avoid complications of conventional surgical techniques such as postsurgical neuralgia, rib resections, muscular atrophy, and trauma [10]. The aim of this study is to assess the feasibility of transesophageal thoracic spine interventions in a porcine model. We report the first transesophageal anterior spinal approach of multiple thoracic vertebrae using NOTES techniques.

## 2. Methods

This study was approved by the University of Puerto Rico Animal Care Institutional Review Board. Acute nonsurvival experiments were performed on four 50 kg pigs (*Sus scrofus domesticus*) under 1.5% to 2% isoflurane general anesthesia with 7.0 mm endotracheal intubation (Mallinckrodt Co, C.D. Juarez, Chihuahua, Mexico). Prior to endoscopy, all pigs were fasted overnight with unrestricted access to water. Preanesthesia medications consisted of an intramuscular injection of 100 mg/mL Telazol (tiletamine HCL + zolazepam HCL; Lederle Parenterals, Inc, Carolina, PR) reconstituted with 100 mg/mL ketamine HCL and 100 mg/mL xylazine at a total dose of approximately 0.05 cc/kg. An intravenous (IV) line (18 g Gelco IV catheter, Medex Inc., Carlsbad, CA) was placed in the marginal ear vein, and 1 g thiopental sodium was injected at a dose of 6.6 to 8.8 mg/kg IV.

A forward-viewing double-channel upper endoscope (GIF-2T160; Olympus Optical Co. Ltd., Tokyo, Japan) was passed perorally and advanced to the esophagus. In pigs, the aortic arch is typically visualized at about 35 cm from the snout and the submucosal tunnel was created starting at approximately 25 cm to facilitate forward viewing of the posterior mediastinum [8]. An initial mucosal incision was created in the right esophageal wall using a Huibregtse single-lumen needle knife (Wilson-Cook Medical Inc., Winston-Salem, NC, USA) with a combination of 20 joules coagulation and 30 joules cutting current (Valleylab SSE2L, Boulder, Col). A submucosal tunnel was created using blunt dissection through the mucosal incision with the tip of a needle knife catheter. The endoscope was introduced into the submucosal space and the channel was extended 5–7 centimeters distally toward the gastroesophageal junction where a full-thickness incision through the muscular layers was completed with a needle knife. The endoscope was passed into the posterior mediastinum and the pig was changed into prone position. Air insufflation was turned off upon entrance into the mediastinum to avoid tension pneumothorax and tension pneumomediastinum while lung ventilation, capnography, pulse oximetry, and heart rate were closely monitored throughout the experiments [3].

The mediastinal compartment, pleura, lung, and the exterior surface of the esophagus were identified immediately after passing the endoscope through the completed myotomy. The anterior thoracic spine, descending aorta, azygous vein, esophagus, chest wall, and superior diaphragmatic surface were examined after placing the pig in prone position and advancing the endoscope in both the forward and retroflexed positions. A lateral decubitus position was evaluated in the process of changing the animals into prone position. However, the fall of the dorsal regions of the lung (lung from the opposite side of decubitus) still interfered with visualization and navigation in the thoracic cavity. Therefore, mediastinoscopy was performed while animals were in prone position. Using the needle knife, small incisions were made through the anterior longitudinal ligament at the level of the proximal, middle, and distal thoracic spine. Vertebral bodies, intervertebral space, and vessels were examined. Vertebral bone biopsy was performed using a 19 gauge needle (Cook Medical, Winston-Salem, NC) or endoscopic biopsy forceps. The needle was advanced into three vertebral bodies (T4, T8, T12) and intervertebral spaces under fluoroscopic monitoring (GE Medical Systems, Milwaukee, WI).

The endoscope was withdrawn from the mediastinum into the esophagus through the submucosal tunnel. The mucosal flap sealed the submucosal tunnel and the mucosal incision was closed with two T-bars (Cook Medical, Winston-Salem, NC). The animals were sacrificed at the end of the procedure for immediate post-mortem examination.

## 3. Results

**Figure 1:**
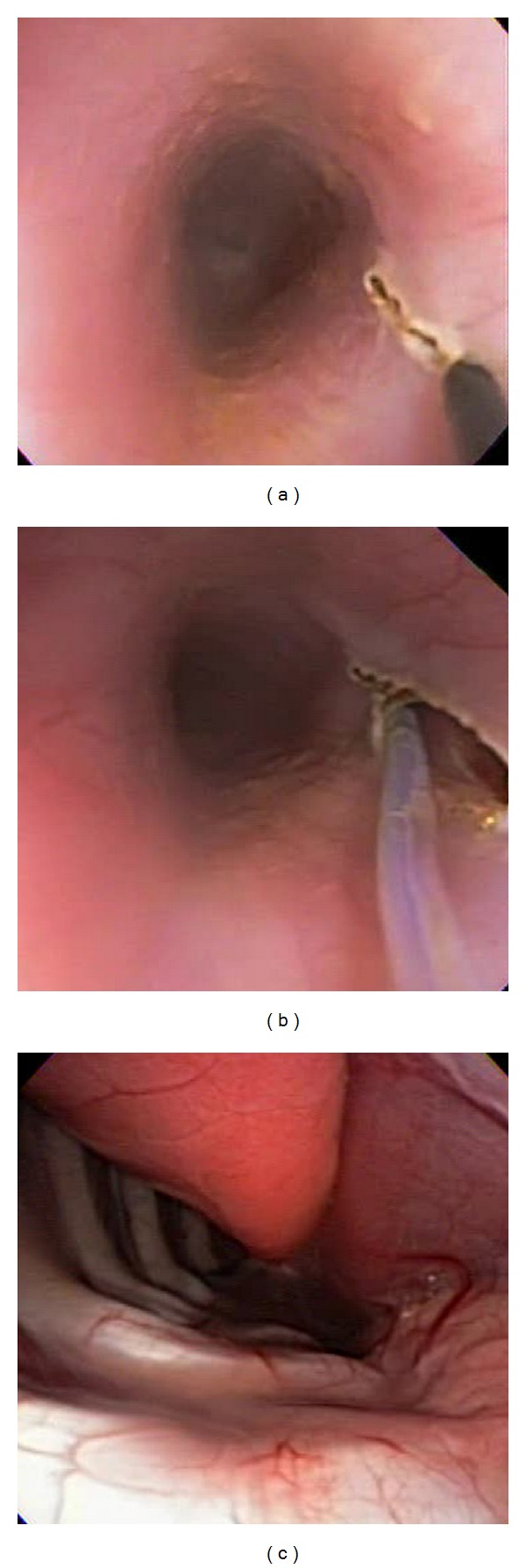
Transesophageal access. (a) Esophageal wall incision. (b) Submucosal tunnel. (c) Visualization of the lung, pleural, aorta, thoracic spine, and esophagus in forward scope position.

We performed acute experiments in four porcine models. Submucosal tunnel was successfully performed in all four pigs as described above and successful access to the mediastinum was attained without injury to any surrounding structures. After passing the endoscope through the completed myotomy, immediate and excellent visualization of lungs, pleura, and margins of the adventitial side of the esophagus were obtained (Figures 1(a)–1(c)). The mediastinal pleura was visualized on each side of the posterior mediastinum overlying the lungs and was not breached. The median time for completion of the transesophageal access was 12 minutes (range, 8–16 minutes).

**Figure 2:**
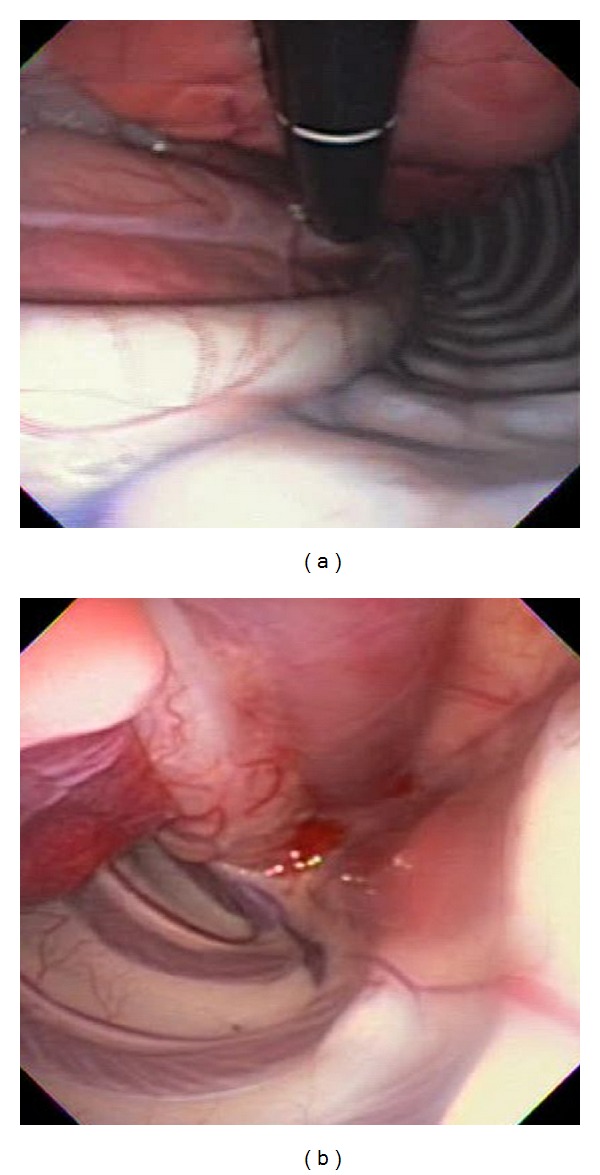
Mediastinoscopy. Retroflexed endoscopic views at (a) distal and (b) proximal thoracic spine.

The posterior mediastinum was evaluated in all animals with no immediate complications. Changing the pig position from supine to prone allowed for spectacular visualization of the entire anterior thoracic spine, descending thoracic aorta, ribs, and the esophagus (Figures 2(a)-2(b)). Further changes in the pigs' position or manipulation of single-lung ventilation were not required to maintain adequate endoscopic visualization during spinal interventions.

**Figure 3:**
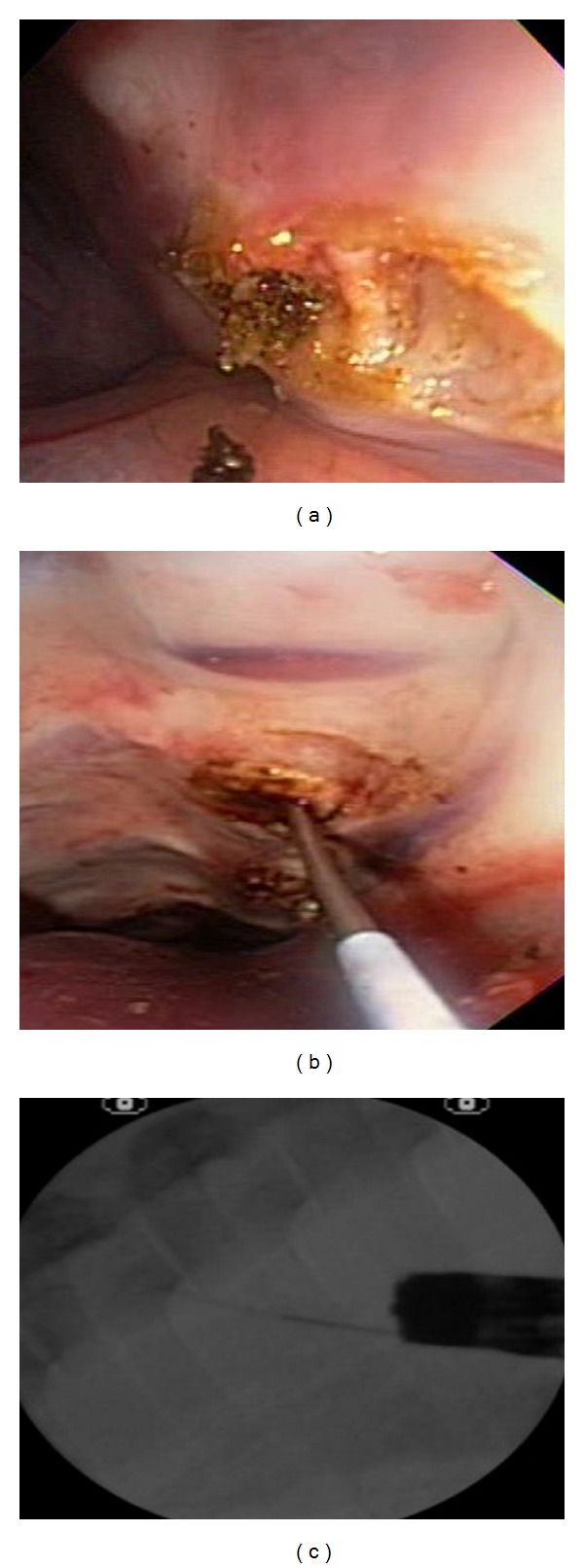
Transesophageal approach to the anterior thoracic spine. (a) Incision over the anterior longitudinal ligament and exposure of the intravertebral space and vertebral bone. (b) Insertion of the 19 gauge needle in the thoracic vertebrae. (c) Fluoroscopic view of vertebral bone biopsy.

Transesophageal interventions in the thoracic spine were successful in all animals. The incision through the anterior longitudinal ligament and subsequent exposure of vertebral bone tissue and intervertebral spaces at the level of the proximal, middle, and distal thoracic spine were successfully completed while avoiding damage to the adjacent vessels. Bone biopsies were successfully obtained from selected thoracic vertebral bodies (T4, T8, T12). Fluoroscopy was used to confirm precise vertebral location. However, the hardness of cortical bone tissue resulted in fragmented bone samples using both forceps and needles, and limited the insertion of the 19 gauge needle to approximately one centimeter into the vertebra as seen under fluoroscopy (Figures 3(a)–3(c)). After 4-5 attempts, the 19 gauge needle was inserted under fluoroscopy guidance approximately one centimeter into the vertebra. Once inserted within the vertebral body and visualized in place under fluoroscopy, the 19 g needle was withdrawn and flushed with water to obtain the specimen. The specimen consisted of fragmented particles (1–3 fragments). These particles were visually inspected and palpated to confirm the presence of bone particles as a measure of sample adequacy (the purpose was to assess the presence or absence of bone material). The median time for entire procedure was 77 minutes (range, 52–93 minutes).

There were no hemodynamic complications during transesophageal access and interventions in the thoracic spine. All animals remained stable throughout the experiment and displayed no changes in hemodynamic parameters or oxygen saturation while completing incisions in the anterior longitudinal ligaments or vertebral bone biopsies. Necropsy revealed no injury to mediastinal organs or vessels resulting from mediastinoscopy, bone biopsy or esophagotomy closure with T-bars. Harvesting of bone fragments was not performed.

## 4. Discussion

Transesophageal NOTES has not garnered as much interest as other approaches for NOTES. There is much more to learn about this technique and its potential applications. The use of a transesophageal NOTES approach for anterior spinal procedures is an innovative technique with the potential for clinical application. Prior experience with submucosal tunneling [8] and peroral endoscopic myotomy (POEM) has suggested safety of such an approach [11, 12]. Access strategies for surgical interventions in the thoracic spine most commonly include thoracotomy, costovertebral, posterolateral, and transpedicular percutaneous approaches [13–16]. Open surgical techniques to expose the spine require the separation of musculoskeletal structures and traction of nerve roots to create an access large enough to accommodate surgical tools. The morbidities associated with these strategies include postsurgical neuralgia resulting from traction injuries to nerve roots, lacerations of the dura mater, scars from skin incisions, wound infection, and muscular atrophy or trauma [16, 17].

Minimally invasive approaches to the thoracic and thoracolumbar spine, such as video-assisted thoracoscopic surgery (VATS), allow the performance of anterior approaches to the spine with small transthoracic incisions or portals [17, 18]. These portals have reduced the size of percutaneous incisions with less muscular dissection and chest wall disruption, all of which contribute to a faster recovery from surgery. Nevertheless, the consequences of a percutaneous access are not totally avoided and patients often require hospital stay following the procedure. Lung atelectasis, empyema, and retropleural effusions are additional morbidities often reported after VATS procedures [18, 19].

The proximity of the esophagus to the vertebral column provides close and direct access to the thoracic spine and opens up new ground for the performance of multilevel anterior spine procedures through NOTES techniques. In this study, the esophageal submucosal endoscopy technique was used to access the posterior mediastinum and to prevent mediastinal soiling in all animals. Although submucosal saline injections or endoscopic mucosal resection (EMR) caps were not utilized, a careful superficial incision in the mucosa followed by blunt dissection of the submucosal layer resulted in a safe entry into the mediastinum with no resulting complications. Selection of the entry site in the right esophageal wall of the proximal to mid esophagus was determined by following known anatomical structures around the esophagus in order to avoid puncture of the aorta or the heart located behind the left or left posterior esophageal wall or the azygous vein behind the right-posterior wall.

Navigation within the thoracic cavity was performed under mechanically-assisted lung ventilation with the endoscopy air pump off. Given that intramediastinal pressures were not monitored, avoiding inadvertent room air insufflation into the thoracic cavity prevented potential complications from positive intramediastinal pressures such as an acute lung or hemodynamic collapse. The gasless approach did not limit access and navigation of the mediastinum or approach to the thoracic spine. It is uncertain if a low-pressure or pressure limited pneumomediastinum could improve exposure even in supine position. This technique could be evaluated in future experiments. More importantly, the use of laparoscopic insufflators for pressure control (intrathoracic pressure monitoring) is an additional safety parameter that must be used in future transesophageal NOTES experiments. None of the animals required intraoperative chest tube placement or suffered cardiovascular complications during the experiment. However, in agreement with other investigators [7, 8], further studies should monitor intrathoracic pressures, ventilation volumes and pressures or insufflation of CO2 as safety parameters while performing transesophageal NOTES interventions in the mediastinum.

Changing the pig position from supine to prone facilitated the visualization of the entire anterior thoracic spine and surrounding structures. Prone position resulted in the fall of the dorsal regions of the lungs into a dependent position away from the vertebral column while keeping both lungs under assisted mechanical ventilation. Adequate visualization was maintained without need for further position changes or single-lung ventilation. In contrast, accessing other areas in the thoracic cavity, such as a left-sided approach to the heart, would still require single-lung ventilation for optimal visualization [7].

In this study, the anterior vertebral bodies and intervertebral spaces were easily approached at different levels of the thoracic spine without injury to the adjacent vessels. Incisions in the anterior longitudinal ligament and vertebral bone biopsy were safely performed under direct endoscopic observation. However, some technical challenges were encountered during vertebral bone biopsy. First, the hardness of the cortical bone of vertebral bodies limited the introduction of the 19-gauge needle to approximately one centimeter into the vertebral bone as observed under fluoroscopy. In addition, the hardness of the cortical bone resulted in small and fragmented tissue samples obtained through both endoscopic forceps and needles. Future development of endoscopic accessories dedicated to bone tissue interventions will be necessary to facilitate sampling or extraction of bone tissue via NOTES techniques. Second, retroflexed position of the endoscope in the posterior mediastinum resulted in a tangential orientation to the spine, which made needle insertion into the vertebral bodies more technically demanding. A side-viewing endoscope can potentially allow an en-face approach to the spine, but this endoscope was not evaluated in the study. In the future, a steerable overtube with variable stiffness technology or a multibending endoscope may reduce tangential orientations and avoid the use of multiple endoscopes in mediastinal NOTES procedures.

A transesophageal approach to the vertebral column has the potential for the development of novel interventions in the anterior thoracic spine under direct endoscopic observation. Examples of these innovative procedures include endoscopic aspiration and biopsy of vertebral bodies when infection or malignant infiltration is suspected and the source of infection or metastasis is unknown; vertebroplasty and kyphoplasty for vertebral compression fractures due to osteoporosis or malignancy; intradiscal therapies such as electrothermal annuloplasty or pulsed radiofrequency ablations for chronic low back pain; and release of the anterior longitudinal ligament at different levels of the vertebral column for severe scoliosis.

The advantages of NOTES for spinal interventions are similar to those of anterior laparoscopic spinal surgery but without the limitations of rigid instrumentation. These benefits include maintenance and ease of restoration of intervertebral disc height, avoidance of removal of bone from the spine, which is an integral component of posterior spinal surgery, and preservation of normal spinal anatomy since this approach takes advantage of normal tissue planes with no removal of bone tissue. In addition, the complications of posterior spinal surgery, such as nerve injury due to manipulation, retraction, and hematoma formation around nerves, and which may cause scarring and chronic pain, can be avoided [20]. Potential disadvantages of transesophageal NOTES include risk of mediastinitis and iatrogenic injury to major vessels and pleura resulting in massive hemorrhage, and tension pneumomediastinum, respectively. Contamination protocols and cultures are a major consideration in spine surgery. Given that the purpose of these nonsurvival experiments was only to assess the feasibility of a transesophageal biopsy of the thoracic vertebrae, infection prevention measures were not followed. Contamination protocols and cultures will be paramount in future survival NOTES experiments in spine surgery.

This initial *in vivo* nonsurvival study reports the first transesophageal intervention in the thoracic spine and proves the feasibility of this novel approach. Esophageal submucosal endoscopy and prone positioning allowed for safe access to the mediastinum and excellent visualization of the vertebral column. The release of the anterior longitudinal ligament, biopsy of multiple vertebral bodies, and exposure intervertebral spaces via NOTES techniques were feasible and safe. The proximity of the esophagus to the vertebral column is favorable for developing novel NOTES spinal interventions.
